# National and International Dimensions of Human Immunodeficiency Virus-1 Sequence Clusters in a Northern California Clinical Cohort

**DOI:** 10.1093/ofid/ofz135

**Published:** 2019-03-14

**Authors:** Soo-Yon Rhee, Brittany R Magalis, Leo Hurley, Michael J Silverberg, Julia L Marcus, Sally Slome, Sergei L Kosakovsky Pond, Robert W Shafer

**Affiliations:** 1 Division of Infectious Diseases, Department of Medicine, Stanford University, California; 2 Department of Biology, Temple University, Philadelphia, Pennsylvania; 3 Division of Research, Kaiser Permanente Northern California, Oakland; 4 Harvard Medical School and Harvard Pilgrim Health Care Institute, Boston, Massachusetts; 5 Department of Infectious Diseases, Kaiser Permanente Northern California, Oakland

**Keywords:** HIV-1, network analysis, pol sequence, transmission

## Abstract

**Background:**

Recent advances in high-throughput molecular epidemiology are transforming the analysis of viral infections.

**Methods:**

Human immunodeficiency virus (HIV)-1 *pol* sequences from a Northern Californian cohort (NCC) of 4553 antiretroviral-naive individuals sampled between 1998 and 2016 were analyzed together with 140 000 previously published global *pol* sequences. The HIV-TRAnsmission Cluster Engine (HIV-TRACE) was used to infer a transmission network comprising links between NCC and previously published sequences having a genetic distance ≤1.5%.

**Results:**

Twenty-five percent of NCC sequences were included in 264 clusters linked to a published sequence, and approximately one third of these (8.0% of the total) were linked to 1 or more non-US sequences. The largest cluster, containing 512 NCC sequences (11.2% of the total), comprised the subtype B lineage that traced its origin to the earliest North American sequences. Approximately 5 percent of NCC sequences belonged to a non-B subtype, and these were more likely to cluster with a non-US sequence. Twenty-two NCC sequences belonged to 1 of 4 large clusters containing sequences from rapidly growing regional epidemics: CRF07_BC (East Asia), subtype A6 (former Soviet Union), a Japanese subtype B lineage, and an East/Southeast Asian CRF01_AE lineage. Bayesian phylogenetics suggested that most non-B sequences resulted from separate introductions but that local spread within the largest CRF01_AE cluster occurred twice.

**Conclusions:**

The NCC contains national and international links to previously published sequences including many to the subtype B strain that originated in North America and several to rapidly growing Asian epidemics. Despite their rapid regional growth, the Asian epidemic strains demonstrated limited NCC spread.

Several phylogeographic studies indicate that human immunodeficiency virus (HIV)-1 subtype B was introduced into the United States from the Caribbean approximately 1970 [1, 2]. In the years since the epidemic was recognized, the subtype B HIV-1 lineage that initiated the US epidemic spread to many other countries [1, 3]. Concurrently, non-subtype-B variants gradually increased in prevalence in the United States [4–6] and other predominantly subtype-B countries [7–10]. By the early 1980s, large numbers of persons suffering from HIV-related illnesses were reported from the New York City, Miami, Los Angeles, and San Francisco metropolitan areas. These regions, frequent destinations for both immigration and travel, were points of origin for the global spread of the subtype B viruses.

Recent advances in high-throughput molecular epidemiology analysis revealed international links to regional HIV-1 epidemics [11]. In this study, we use a large collection of HIV-1 *pol* sequences in a Northern Californian cohort sampled over 2 decades, and we published *pol* sequences from other parts of the United States and from outside of the United States to quantify the degree and nature of national and international links in this cohort.

## METHODS

### Persons and Virus Sequences

We analyzed protease (PR) and reverse-transcriptase (RT) sequences from a cohort of antiretroviral therapy (ART)-naive persons from the Kaiser Permanente Medical Care Program-Northern California (KPNC) undergoing genotypic resistance testing at Stanford University between 1998 and 2016 (Northern California cohort [NCC]). The KPNC is estimated to provide care to approximately 30% of the insured population in Northern California. Genotypic resistance testing for ART-naive individuals became routine in 2003 [12, 13]. Protease and reverse-transcriptase nucleotide sequences from 4553 individuals in the Northern California cohort are available in GenBank and the accession numbers are listed in the Supplementary Files.

We searched the Los Alamos National Laboratories (LANL) HIV Sequence Database, which contains all published HIV-1 sequences, to identify sequences encompassing PR and at least the first 200 amino acids of RT (HXB2 nucleotides 2253–3151) [14]. When more than 1 sequence from an individual is available, the representative sequence was selected at random per LANL protocols. When more than 1 sequence was available for persons in the NCC, we selected the earliest sequence. All LANL sequences from individuals also in the NCC were excluded from the LANL dataset. Sequences annotated as problematic by LANL were also excluded: synthetic sequences and those with high non-ACTG content, hypermutation, and potential contamination [14, 15].

The LANL reference dataset, generated July 1, 2018, included 139 060 HIV-1 group M sequences. The LANL and NCC sequences were annotated with subtype, country, sample year, and surveillance drug-resistance mutations (DRMs) [16]. The NCC sequences were subtyped using the COMET program [17]. The NCC sequences were also annotated with age, gender, race, and HIV acquisition risk factor. Within the NCC, a generalized binomial logistic regression model was used to assess the relationship between sample year and proportion of persons with a non-B subtype.

### Transmission Network Analyses

The HIV-TRAnsmission Cluster Engine (HIV-TRACE) was used to infer a molecular transmission network designed to identify NCC sequences genetically similar to sequences in the LANL dataset [18]. The NCC and LANL sequences were aligned to a reference PR and RT sequence (HXB2; GenBank accession no. K03455) using the codon-aware program *bealign* (BioExt package, https://github.com/veg/BioExt). Tamura-Nei (TN93) pairwise nucleotide genetic distances were calculated between each pair of sequences in the combined datasets, and sequence pairs with TN93 distances ≤2% were recorded for subsequent analyses [18, 19]. Ambiguous nucleotides were handled as described previously by resolving 2-way ambiguities (RYMSWK) to maximize matches, averaging all other ambiguities, and averaging all ambiguities in sequences that contained 5% or more of ambiguous nucleotide positions [18]. A phylogenetic test of conditional independence was used to remove some spurious transitive connections that result in cycles of transmission using an edge filtering method (described in Supplementary Methods).

We used a 2-tiered TN93 distance cutoff to define a link (edge) between 2 sequences (nodes): a 1.5% threshold for linking NCC sequences to other NCC sequences and to LANL sequences [18], and a more stringent 1.0% to bring in LANL sequences that connect only to other LANL sequences [19]. The NCC transmission network contained clusters having 2 or more NCC sequences. The NCC-LANL transmission network contained clusters having 1 or more NCC sequences linked to 1 or more LANL sequences.

### Phylogenetic Analyses

Phylogenetic and molecular clock analyses were performed to characterize the phylogenetic structure and transmission characteristics of several of the largest clusters. Maximum likelihood (ML) trees were constructed in IQ-TREE version 1.6.5 using the best-fitting nucleotide substitution model according to Bayesian information criterion [20, 21]. Evidence in support of the use of a molecular clock model was assessed in TempEst version 1.5.1 using linear regression of the relationship of root-to-tip genetic divergence from the best-fitting root within the maximum likelihood tree of each taxon with sampling time [22]. Regression analysis revealed a positive rate of genetic divergence and random residual pattern for each of the 4 major clusters, indicating sufficient measurable evolution among the sequenced cohort for divergence dating (Supplementary Figure 1). Bayesian phylogenetic inference of time-scaled trees was performed using Markov chain Monte Carlo (MCMC) sampling implemented in BEAST version 1.10 using parameters described in the Supplementary Methods [23, 24]. To accelerate molecular clock analyses on clusters with large numbers of sequences, we systematically subsampled the sequences from the larger clusters using a principled heuristic described in the Supplementary Methods.

## RESULTS

### Persons and Virus Sequences

Between 1998 and 2016, 4553 ART-naive NCC members underwent genotypic resistance testing. The median sample year was 2009 (interquartile range [IQR], 2006–2013); 90.0% underwent testing after 2003 when pretherapy genotypic resistance testing became routine. Approximately 91% were male and the median age was 39 (IQR, 31–48). The racial/ethnic composition was 46.3% white, 22.4% African American, 20.8% Latino, 8.7% Asian, 0.4% American Indian/Alaska Native, and 1.5% unknown. The HIV-1 acquisition risk factors included men who have sex with men ([MSM] 69.9%), heterosexual contact (20.3%), intravenous drug use (3.8%), transfusion (0.4%), and unrecorded (5.6%).

Subtype B accounted for 95.5% of HIV-1 isolates from NCC members (n = 4350). The remaining 4.5% (n = 203) belonged to a non-B subtype: C (n = 55), CRF01_AE (n = 52), CRF02_AG (n = 24), A (n = 15) and other miscellaneous subtypes, circulating and unique recombinant forms (n = 57). There was a statistically significant increase in non-B sequences prevalence from 1.8% between 1998 and 2003 to 5.5% between 2011 and 2016 (odds ratio, 1.06; 95% confidence interval [CI], 1.03–1.10).

### The Transmission Network

**Figure 1.**
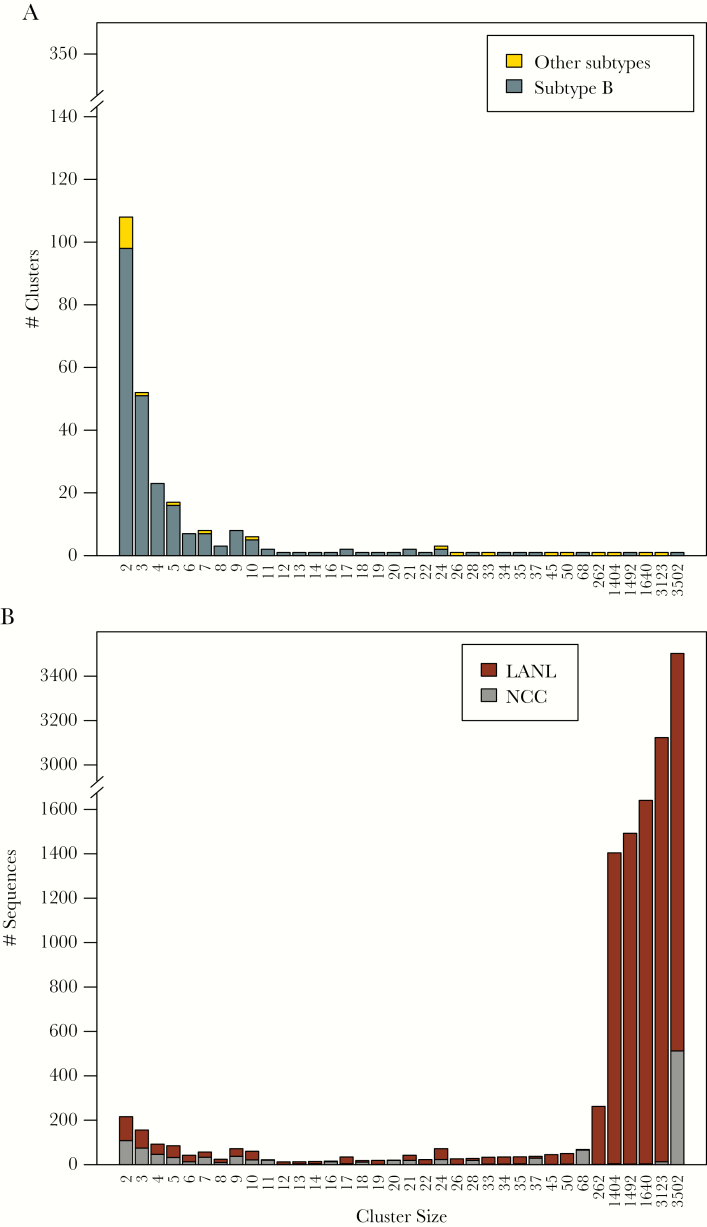
Distribution and composition of cluster sizes in the Northern California Cohort (NCC) plus Los Alamos National Laboratories (LANL) transmission network. Figure 1A shows the numbers of clusters according to their sizes. Subtype B sequences are indicated in dark gray and non-subtype B sequences are indicated in yellow. Figure 1B shows the counts of NCC and LANL sequences in clusters grouped by their sizes with NCC sequences indicated in gray and LANL sequences indicated in dark red.

The inferred transmission network contained 679 clusters ranging in size from 2 to 3502 sequences including 415 clusters containing just NCC sequences and 264 clusters containing both NCC and LANL sequences. The 415 NCC-only clusters comprised 1240 NCC sequences (27.2% of all NCC sequences). The 264 NCC-LANL clusters included 1151 NCC sequences (25.3% of all NCC sequences) and 11 735 LANL sequences (8.4% of all LANL database sequences). Overall, 2391 of 4553 NCC sequences (52.5%) were in a cluster, and 1944 (81.3%) of those were directly linked to other NCC sequences. The majority (95.4%) of clusters contained 2 to 13 sequences, with 31 large clusters containing 14 or more sequences. Of the 28 largest clusters with both NCC and LANL sequences, 9 contained non-B viruses (Figure 1A), and most contained primarily LANL sequences (Figure 1B).

The distribution of TN93 distances for edges connecting NCC sequences to one another (median, 0.85%; IQR, 0.50%–1.19%) was significantly lower than the distribution of distances for edges connecting NCC and LANL sequences (median, 1.35%; IQR, 1.19%–1.44%; *P* < .001, 2-tailed Wilcoxon rank-sum test). The median difference in sample years between linked NCC and LANL sequences was 2 years (IQR, 1–4) for sequence pairs with a TN93 distance up to 0.5%, 4 years (IQR, 2–7) for sequence pairs with a genetic distance between 0.5% and 1.0%, and 6 years (IQR, 3–14) for sequence pairs with a genetic distance greater 1.0% (Supplementary Figure 2).

**Figure 2.**
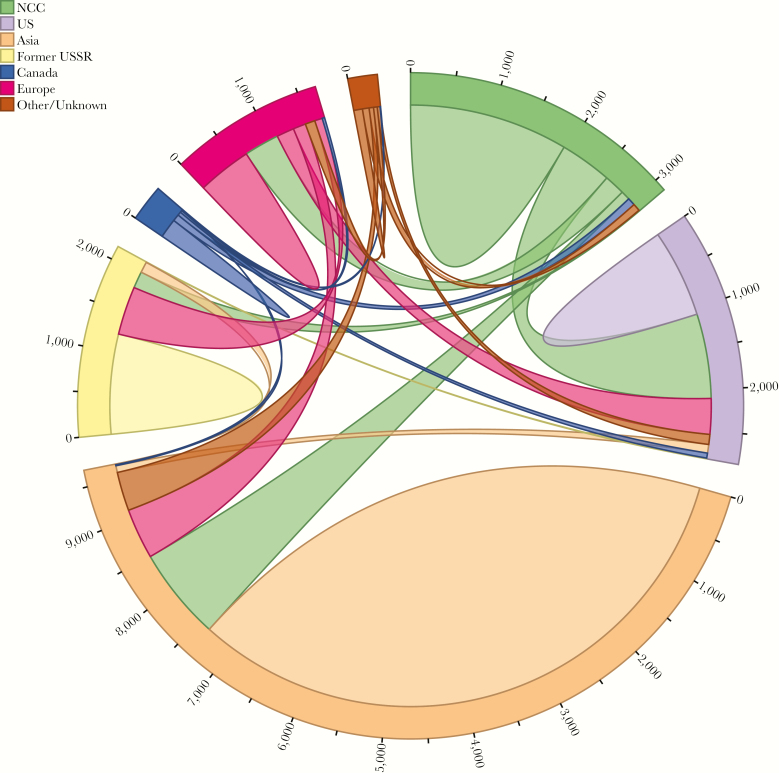
Connection patterns between Northern California Cohort (NCC) and Los Alamos National Laboratories sequences, categorized by country/region of origin. Sequences connected to multiple countries/regions are represented once for each connection.

Of the 1151 sequences in the 264 NCC-LANL clusters, 791 (68.7%) were directly linked to a LANL sequence, whereas 360 (31.3%) were directly linked only to other NCC sequences. Of the 791 NCC sequences directly linked to an LANL sequence, 425 (53.7%) linked solely to 1 or more US sequences, 156 (19.7%) linked solely to 1 or more non-US sequences, and 210 (26.5%) linked to both US and non-US sequences. The intricate web of interconnectivity between the NCC and LANL sequences is illustrated in Figure 2.

The 326 clusters with 3 or more sequences were further categorized into broad geographic classes: 223 (68.4%) local clusters (>60% of cluster members were from NCC), 50 (15.3%) national clusters (>50% of cluster members were from the United States, but not from NCC), and 33 (10.1%) international (>50% of cluster members were from outside the United States). Twenty (6.13%) clusters with 3 or more sequences did not belong to 1 of the 3 classes. The 1151 NCC sequences clustering with an LANL sequence were significantly more likely than the 3402 that did not cluster to be from persons who were male (94.4% vs 89.3%; *P* < .001), younger (median 38 vs 40 years; *P* < .002), MSM (74.7% vs 68.3%; *P* < .001), and intravenous drug users (6.8% vs 3.5%; *P* < .001). Within the clusters containing 3 or more sequences, NCC persons in the international clusters were more likely to be older (median, 41 vs 34 years; *P* < .001), white (53.8% vs 43.5%; *P* < .001), and female (8.3% vs 4.8%; *P* < .001) and less likely to be MSM (71.2% vs 78.5%; *P* < .001) compared with those in local and national clusters.

### Subtype B and Non-B Sequences

Of the 203 NCC non-B sequences, 57 (28.1%) were directly linked to other NCC sequences and 45 (22.2%) were clustered with 1 or more LANL sequences including 41 directly linked to an LANL sequence. Of these 41 sequences, 33 (80.5%) were directly linked to a sequence in 1 or more non-US countries. The proportions of NCC sequences directly linked to LANL sequences was not significantly different between subtype B and non-B sequences (17.2% vs 20.2%; *P* = .32, χ^2^ test). Of the 23 non-B clusters with LANL sequences, 16 included 1 or more sequences from outside of the United States (Table 2). Compared to the 158 NCC non-B sequences that did not cluster with LANL sequences, the 45 NCC sequences that did cluster were significantly more likely to be from persons who were male (77.8% vs 58.9%; *P* = .032), Asian (40.0% vs 17.7%; *P* = .003), MSM (44.4% vs 26.6%; *P* = .035), or intravenous drug users (6.7% vs 0%; *P* = .01) and less likely to be African American (22.2% vs 54.4%; *P* < .001).

Of the 264 NCC-LANL clusters within the inferred transmission network, 241 (91.3%) contained subtype B sequences and 23 (8.7%) contained non-B subtype sequences including 7 CRF01_AE clusters, 3 subtype C clusters, 2 subtype D clusters, and 11 additional clusters each containing sequences belonging to a different subtype, circulating recombinant form (CRF), or unique recombinant form. Table 1 summarizes the sizes and spatiotemporal characteristics of the 28 subtype B clusters with 10 or more sequences. Table 2 summarizes the sizes and the spatiotemporal characteristics of the 23 non-B clusters.

**Table 1.** 28 Subtype B Clusters Containing 10 or More Virus Sequences, and Including Both NCC and LANL Sequences

Size	Class	NCC^a^	LANL Direct^b^	LANL Indirect^c^	Median Year NCC^d^	Median Year LANL^d^	Countries (LANL)
3502	International	512	1172	1818	2008	2003	USA (1122), China (638), Canada (248), Japan (241), Switzerland (178), Italy (91), and 38 other countries, and 1 with no country data
1492	International	2	239	1251	2007.5	2010	Japan (1471), UK (13), USA (2), China (2), Germany (1), Hong Kong (1)
68	Local	65	3	0	2010	2009	USA (3)
37	Local	29	7	1	2007	2008.5	USA (7), Hong Kong (1)
35	National	3	13	19	2006	2004.5	USA (32)
34	National	2	7	25	2008.5	2004	USA (20), Canada (12)
28	Local	19	8	1	2008	2005	USA (6), Canada (2), Japan (1)
24	Local	21	3	0	2013	2005.5	USA (3)
24	International	1	3	20	2013	2011	Philippines (12), Canada (3), Thailand (2), Taiwan (2), Australia (1), Brazil (1), and 2 other countries
22	International	1	6	15	2014	2004	Denmark (13), Sweden (7), Norway (1)
21	Local	18	3	0	2015	2010	USA (3)
21	International	1	1	19	2005	2009.5	Japan (20)
20	Local	19	1	0	2009	2009	USA (1)
19	International	1	4	14	2007	2005.5	UK (11), Australia (4), Singapore (1), Canada (1), USA (1)
18	Local	11	6	1	2006	1999	USA (7)
17	International	3	7	7	2010	2009	Canada (13), USA (1)
17	International	1	13	3	2011	2010.5	Poland (7), UK (6), USA (2), Germany (1)
16	Local	13	2	1	2012	2006	USA (3)
14	National	4	2	8	2011	2002.5	USA (10)
13	National	3	5	5	2008	2005	USA (10)
12	International	1	2	9	2013	2008	UK (5), Germany (2), USA (1), Spain (1), Canada (1), Serbia (1)
11	Local	10	1	0	2013	2007	USA (1)
11	Local	10	1	0	2007.5	2011	Germany (1)
10	Local	9	1	0	2008	2008	USA (1)
10	National	4	6	0	2005.5	2003	USA (6)
10	N/A	4	5	1	2015	2011	USA (5), Australia (1)
10	National	2	6	2	2004	2009	USA (7), Italy (1)
10	National	1	8	1	2007	2005	USA (9)

Abbreviations: LANL, Los Alamos National Laboratories; N/A, nonapplicable; NCC, Northern California Cohort.

^a^Number of sequences from the NCC.

^b^Number of previously published sequences in the Los Alamos National Laboratories HIV Sequence Database (LANL) directly linked (TN93 genetic distance ≤0.015) to an NCC sequence in the cluster. ^c^Number of LANL sequences in the cluster directly linked only to another LANL sequence in the cluster. ^d^Median Isolation year for the NCC and LANL sequences.

**Table 2.** 23 Clusters With Non-B Subtype, and Including Both NCC and LANL Sequences

Subtype	Class	Size	NCC^a^	LANL Direct^b^	LANL Indirect^c^	Median Year NCC^d^	Median Year LANL^d^	Countries (LANL)
01_AE	International	3123	13	58	3052	2010	2009	China (2093), Vietnam (564), Thailand (179), Japan (95), Czechia (39), Australia (21), and 20 other countries, and 7 with no country data
07_BC	International	1640	3	727	910	2014	2011	China (1619), Japan (10), Hong Kong (3), Poland (1), Australia (1), UK (1), and 2 other countries
A6	International	1404	4	250	1150	2009	2005	Russian Federation (703), Uzbekistan (124), Ukraine (117), Latvia (99), Kazakhstan (83), Czechia (60), and 22 other countries
55_01B	International	262	1	1	260	2015	2011	China (258), Thailand (1), Hong Kong (1), Japan (1)
C	International	50	2	39	9	2006	2008	India (34), China (8), Italy (1), Nepal (1), Thailand (1), Czechia (1), and 2 other countries
BF1	International	45	1	10	34	2014	2013	Turkey (42), Cyprus (1), Sweden (1)
24_BG	International	33	2	7	24	2013	2003	Cuba (29), Spain (2)
51_01B	International	26	1	1	24	2011	2013	Mongolia (19), Singapore (4), Thailand (1), Canada (1)
01_AE	International	24	1	17	6	2010	2008	Japan (6), Australia (5), Malaysia (3), Republic of Korea (2), Singapore (1), Belgium (1), and 3 other countries, and 2 with no country data
G	National	10	1	2	7	2016	2011	USA (9)
D	N/A	7	4	3	0	2005	1997	Uganda (3)
01_AE	N/A	5	1	4	0	2008	2010	Hong Kong (1), Philippines (1), and 2 with no country data
02_AG	National	3	1	2	0	2012	2009	USA (2)
01_AE	N/A	2	1	1	0	2008	2009	USA (1)
F1	N/A	2	1	1	0	2014	2013	Spain (1)
01_AE	N/A	2	1	1	0	2016	2013	Philippines (1)
01_AE	N/A	2	1	1	0	2007	2008	Thailand (1)
D	N/A	2	1	1	0	2006	2002	USA (1)
BG	N/A	2	1	1	0	2011	2009	USA (1)
C	N/A	2	1	1	0	2004	2008	USA (1)
01_AE	N/A	2	1	1	0	2011	2013	Philippines (1)
08_BC	N/A	2	1	1	0	2012	2012	China (1)
C	N/A	2	1	1	0	2014	2008	USA (1)

Abbreviations: LANL, Los Alamos National Laboratories; NA, nonapplicable; NCC, Northern California Cohort.

^a^Number of sequences from the Northern California Cohort (NCC).

^b^Number of previously published sequences in the LANL HIV Sequence Database directly linked (TN93 genetic distance ≤0.015) to an NCC sequence in the cluster.

^c^Number of LANL sequences in the cluster directly linked only to another LANL sequence in the cluster.

^d^Median isolation year for the NCC and LANL sequences.

### Sequences With Drug-Resistance Mutations

In 27 (10.2%) of the 264 NCC-LANL clusters, 42 NCC sequences shared 1 or more established DRMs with a linked LANL sequence; 11 of these sequences shared 2 or more DRMs between the NCC and LANL sequences. Eighteen sequences had shared nonnucleoside reverse-transcriptase inhibitor (NNRTI) resistance, 17 had shared nucleoside reverse-transcriptase inhibitor (NRTI) resistance, 4 had shared protease inhibitor (PI) resistance, and 3 had shared dual NRTI/PI resistance. Forty of the 42 sequences with shared DRMs were from the United States and 2 were from Europe; 40 comprised subtype B viruses. The most common shared mutations within these sequences were the NRTI-resistance mutations T215D/E/S/C/V (18 sequences) and M41L (11 sequences); the NNRTI-resistance mutations K103N (12 sequences) and Y188L (3 sequences); and the PI-resistance mutations L90M (4 sequences) and I54V (3 sequences).

### Notable Clusters and Northern California Cohort-Associated Transmission Events

The largest subtype B cluster contained 3502 sequences including 512 NCC sequences and 2990 LANL sequences from 45 countries, including 1868 from outside the United States. Seven of the US sequences were isolated in 1978 and 1979, suggesting that this cluster originated from the founding viruses of the US subtype B epidemic [2]. Twenty-five NCC sequences shared a TN93 genetic distance ≤1.5% with 1 of the 7 1978/1979 US subtype B founder viruses. This cluster represents a lineage that originated in Haiti and the United States and then spread widely to many countries in Europe, Asia, and South America [1, 3, 25, 26], which explains its designation as an international cluster. The overall mean distance between sequence pairs in this cluster was 3.5% with the maximum distance between sequence pairs of 9.4%. The large size of the cluster likely results from chaining caused by the dense sampling of viruses belonging to this lineage since the earliest of these strains were identified 35 years ago.

The second largest subtype B cluster contained 1492 sequences including 2 NCC sequences and 1490 LANL sequences, 1471 of which were from Japan. This cluster contained sequences closely related to the previously reported JP.MSM.B-1 strain, which accounts for one third of infections in Japanese MSM [27] (Supplementary Figure 3). One of the 2 NCC sequences was found within a well supported subclade originating in 1995 (approximate high posterior density [HPD] interval: 1992–1998) (Supplementary Figure 4). This subclade contained 4 European and 4 Asian sequences described by Takebe et al [27] as evidence of global dissemination of the B-1 strain.

**Figure 3.**
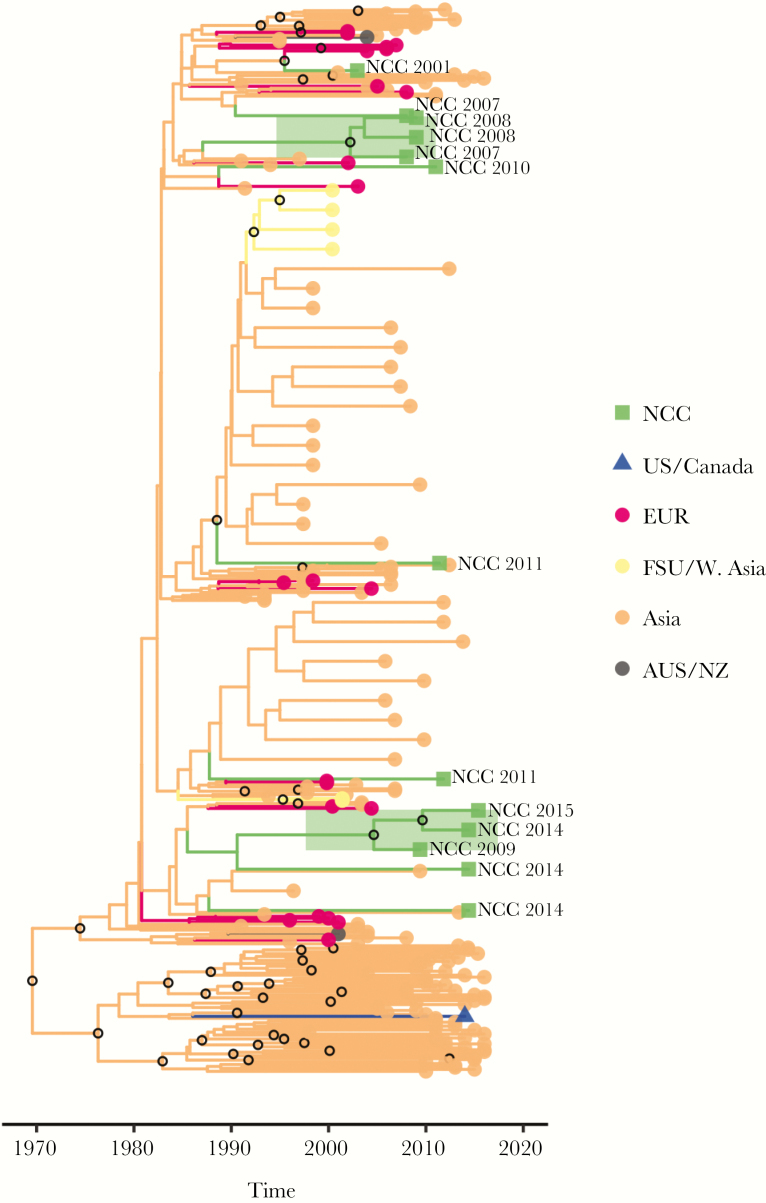
Bayesian maximum clade credibility tree for the largest CRF01_AE cluster. Branch lengths are scaled in time and are colored according to region (legend at right) determined using maximum parsimony ancestral state reconstruction. Clades containing Northern California Cohort (NCC) sequences are expanded for clarity, and the 2 highly supported (posterior probability [PP] ≥90%) clades comprised of NCC sequences only are highlighted in green. In addition, the location of NCC sequences is indicated by taxon labeling. Open circles located at interior nodes indicate PP ≥90%.

The largest non-B cluster contained 3123 CRF01_AE sequences including 13 NCC sequences, 58 LANL sequences directly linked to 1 of these 13 sequences, and 3052 additional LANL sequences. Most of the sequences in this cluster were from China, Vietnam, Thailand, or Japan. Two subclades involving 3 NCC lineages each provide evidence of at least 2 separate ongoing transmission chains within the NCC, originating in 2003 (HPD: 2001–2006) and 2006 (HPD: 2004–2009) (Figure 3).

The second largest non-B cluster contained 1640 CRF07_BC sequences including 3 NCC sequences, 727 LANL sequences directly linked to 1 of these 3 NCC sequences, and 910 additional LANL sequences. Most (1619) of these sequences were from China (Table 2). The recent origin of this cluster (1990; HPD, 1984–1995) is consistent with previous descriptions of this subtype as a rapidly growing population [28]. There is no evidence for ongoing transmission of these viruses in the NCC (Supplementary Figure 5).

The third largest non-B cluster included 1404 subtype A6 sequences including 4 NCC sequences, 250 LANL sequences directly linked to 1 of these 4 sequences, and 1150 additional LANL sequences [29]. Most of these sequences were from countries of the former Soviet Union (FSU) (Table 2). Molecular dating of this cluster using relaxed clock methods proved difficult. Bayesian estimates of cluster tMRCA were sensitive to the model of population size dynamics and showed wide CIs (1939; HPD, 1901–1969). Our analyses included many sequences outside the FSU, hence its results are not directly comparable to previous, FSU-focused, estimates. No significant evidence of onward transmission was found within the NCC (Supplementary Figure 6).

Several additional examples of how the inclusion of published sequences provides insight into transmission dynamics are shown in Supplementary Figure 7. For example, a cluster of just 2 CRF24_BG NCC sequences was expanded to a cluster of 33 sequences involving 2 additional countries (Supplementary Figure 7A). Two NCC subtype C sequences that were disconnected in the NCC-only network became hubs in a cluster of 50 sequences from 7 additional countries (Supplementary Figure 7B). A cluster of 2 NCC subtype B sequences and an unconnected subtype B NCC sequence were linked to a cluster of LANL isolates from the United States and Canada (Supplementary Figure 7C), and a cluster of 4 NCC sequences was augmented with a LANL US isolate and bridged to an older cluster of LANL sequences (Supplementary Figure 7D).

## DISCUSSION

Standard practice in molecular epidemiology inference is to consider only sequences from a local or regional epidemic or to include a small subset of preselected reference sequences [30, 31]. By expanding our analysis to all previously published sequences, we analyzed our local epidemic within the context of the global HIV-1 pandemic. Twenty-five percent of 4553 NCC sequences were in a cluster with 1 or more previously published sequences, and more than two thirds of these (17.4% of the total) had a direct link, defined as genetic distance ≤1.5%, to 1 or more previously published sequences. Approximately one half of these linked sequences (8.0% of the total) were linked to 1 or more non-US sequences.

Subtype B accounted for 95.5% of the NCC sequences. The largest subtype B cluster contained many NCC sequences consistent with the fact that this lineage traces its origin to the United States [26]. Although most of the other large subtype B clusters were dominated by US sequences, the second largest subtype B cluster, which contained 2 NCC sequences, traced its origin to Japan.

Non-B subtypes accounted for 4.5% of the NCC sequences, and, unsurprisingly, these were more likely to cluster with a non-US sequence. The CRF07_BC cluster, which originated in East Asia, contained 3 NCC sequences, each of which appeared to represent a separate NCC introduction. The subtype A6 cluster, which originated in countries of the former Soviet Union, contained 4 NCC sequences, each of which also appeared to represent separate NCC introductions. In contrast, the largest CRF01_AE cluster, which was dominated by sequences from East and Southeast Asia, contained 13 NCC sequences, including 2 lineages, each containing 3 sequences, consistent with instances of ongoing local transmission. Although 2 of the 3 NCC individuals in the CRF07_BC cluster and 8 of the 13 NCC individuals in the largest CRF01_AE cluster were of Asian ethnicity, the countries of origin of NCC individuals were not available. The absence of data on country of origin is a limitation of our study that makes it impossible to distinguish between immigration and local dissemination as a cause of non-subtype B diversity in our cohort.

Approximately 1% of NCC sequences shared 1 or more established DRMs with a previously published sequence. The vast majority were subtype B sequences from other persons in the United States, consistent with the high prevalence of transmitted drug resistance (TDR) in the United States. However, the proportion of NCC TDR sequences linked to TDR sequences outside of the NCC was much lower than the 37% of NCC TDR sequences previously reported to link to 1 or more other NCC TDR sequences [12].

Despite a statistically significant positive correlation between the genetic distance between sequences and the length of time separating their isolation dates, there were many exceptions to this trend. Indeed, even sequences that differ from one another by ≤0.5% were often obtained several years apart, demonstrating that the presence of highly similar sequences between a pair of individuals should not be interpreted as evidence for direct transmission. Hypotheses about direct transmission make sense only when sequence analysis is combined with contact tracing [32–34].

As the number of sequences in transmission network analyses have risen, it has become increasingly common to first identify clusters using a rapid distance-based approach and to then use phylogenetic analysis to study individual clusters [19, 35]. Because of how HIV-TRACE defines clusters, adding more sequences to the sample set can create new clusters, merge existing clusters, or add sequences to existing clusters. In contrast, clusters that are defined phylogenetically may disappear entirely with the addition of more sequence data, because, for example, the bootstrap support for subtending branches is degraded [36].

Including the totality of publicly available sequences in our analysis had several measurable and important benefits. First, by comparing NCC with previously published sequences, an additional 429 NCC sequences (9.4% of the total) were brought into the network, increasing the total number of clustered NCC sequences to 2391 or 52.5% of the total. This rate is similar to the rate of 52.3% reported for a smaller and intensively sampled cohort from San Diego [37], and it is notably higher than the rate of 32% reported from US national surveillance data [38]. In addition, the placement of individual sequences in the clusters was altered by the inclusion of previously published sequences, with NCC sequences becoming peripheral both in the large non-subtype B clusters displaying patterns of exponential growth and in many of the smaller clusters. Because HIV transmission dynamics can be strongly influenced by central nodes in transmission networks (and that there are relatively few of them), being able to more accurately identify such nodes is also important [39].

## CONCLUSIONS

In conclusion, the NCC epidemic is dominated by subtype B viruses linked primarily to other NCC sequences. However, the largest subtype B cluster, which retained links to the earliest North American subtype B strains, contained large numbers of links to persons in other parts of the United States, Asia, and Europe. Several sequences belonged to well characterized international clusters responsible for rapidly expanding regional epidemics. However, despite their rapid regional spread, these viruses do not appear to have made significant inroads into the NCC, suggesting that HIV-1 transmission is influenced more by human behavioral factors than intrinsic viral factors. Continued monitoring of local epidemics in the context of global sequences will advance a better understanding the past, present, and potentially future courses of regional epidemics and support public health measures to optimally target prevention efforts.

## Supplementary Data

Supplementary materials are available at *Open Forum Infectious Diseases* online. Consisting of data provided by the authors to benefit the reader, the posted materials are not copyedited and are the sole responsibility of the authors, so questions or comments should be addressed to the corresponding author.

ofz135_suppl_Supplementary_Materials
