# Supplementary material for: National and International Dimensions of Human Immunodeficiency Virus-1 Sequence Clusters in a Northern California Clinical Cohort
Source: Open Forum Infect Dis. 2019 Mar 14;6(4):ofz135. doi: 10.1093/ofid/ofz135 (PMC6483754; doi:10.1093/ofid/ofz135)
Supplement: ofz135_suppl_Supplementary_Materials [file ofz135_suppl_supplementary_materials.docx]

**TITLE**

National and International Dimensions of HIV-1 Sequence Clusters in a Northern California Clinical Cohort

**Running TITLE**

U.S. and Global HIV-1 Sequence Clusters

**AUTHORS**

Soo-Yon Rhee^1*^, Brittany R. Magalis^2*^, Leo Hurley^3^, Michael J. Silverberg^3^, Julia L. Marcus^4^, Sally Slome^5^, Sergei L. Kosakovsky Pond^2†^, Robert W. Shafer^1†^

^1^Division of Infectious Diseases, Department of Medicine, Stanford University, CA, U.S.A.

^2^Department of Biology, Temple University, Philadelphia, PA, U.S.A

^3^Division of Research, Kaiser Permanente Northern California, Oakland, CA, U.S.A.

^4^Harvard Medical School and Harvard Pilgrim Health Care Institute, Boston, MA, U.S.A.

^5^Department of Infectious Diseases, Kaiser Permanente Northern California, Oakland, CA, U.S.A.

**Supplementary Methods**

**Edge filtering**

The phylogenetic test of conditional independence considers all triangles in the network and determines whether or not a triangle (which implies a biologically implausible circular transmission patterns) can be simplified into a transmission chain. If we consider a phylogenetic tree on three sequences, then the situation where one of the sequences is not significantly different from the root sequence (tested via a likelihood ratio test in the standard phylogenetic maximum likelihood framework, where the null hypothesis is that a specific branch length is 0, and the alternative is that it is different from 0) implies that the other two sequences can be viewed as phylogenetically independent from each other given the root sequence (see Supplementary Figure 8). In that case a transmission chain where the middle sequence is the root sequence can be used to replace the triangle. Edge filtering on the entire network proceeds as follows.

1. Enumerate all triangles (subject to an upper bound of 218 for computational) in the network.
2. For each triangle perform the phylogenetic test. If more than one branch is not significantly different from zero, choose one with the least support (as measured by the highest p-value); break ties randomly.
3. Rank all the network edges marked for removal by triangle tests using the following heuristics:
4. Never remove edges that would disconnect existing clusters.
5. The edges with the highest p-values (least support) are removed first.
6. Iterate 1-3 until no edges are removed.

This procedure will not change the number of clusters by design, but it does remove a fair number of links from the network (~20%).

**Subsampling sequences from a larger cluster**

To accelerate these analyses on clusters with large numbers of sequences, we subsampled the sequences from the larger clusters using a heuristic that retained all NCC sequences and all those LANL sequences that were directly linked them at a TN93 distance ≤0.5%. Next, we thinned out highly similar groups of sequences by partitioning LANL sequences into completely linked subclusters having diameter of ≤0.5% TN93 distance. From each subcluster we sampled one sequence per represented calendar year, thereby ensuring maximal temporal coverage.

**Phylogenetic inference of time-scaled trees**

Maximum likelihood (ML) trees used for linear regression analysis of measurable evolution were reconstructed in IQ-TREE v1.6.5 using the best-fitting nucleotide substitution model according to Bayesian information criterion [1, 2]. Bayesian phylogenetic inference of time-scaled trees was performed using Markov chain Monte Carlo (MCMC) sampling implemented in BEAST v1.10 [2, 3] assuming the uncorrelated, relaxed molecular clock [1]. Marginal likelihood estimates using stepping stone analysis [4] were obtained for the constant, exponential, and Bayesian Skyride (non-parametric) demographic models [5] in order to determine the best-fitting model of change in effective population size over time. Independent MCMC analyses were run for 100 million generations or until effective sample sizes reached values greater than 200 after burn-in of 10% of samples [6]. Three of the major clusters (A6, B (JP), and CRF01_AE) were best explained by a non-parametric model, whereas cluster CRF07_BC was best explained by exponential growth, with ln(Bayes Factor) > 10 indicative of strong support for the alternative model (Table S1). Results of the MCMC analysis were summarized within the maximum clade credibility trees for each cluster.

**Data availability**

Relevant data, including but not limited to, sequence alignments and trees, as well as detailed methodology are available from <https://github.com/veg/kpnet-global>.

**Supplementary Table 1**

| Table S1. Marginal likelihood estimates for demographic models using stepping stone sampling. | | | | |
| --- | --- | --- | --- | --- |
| **Model** | **Cluster** | | | |
|  | **B (JP)** | **CRF01_AE** | **CRF07_BC** | **A6** |
| **Constant** | -15570.23 | -17664.37 | -13554.03 | -14816.94 |
| **Exponential** | -15474.96 | -17553.37 | **-13289.57** | -15440.57 |
| **Skyride** | **-15459.60** | **-17343.39** | -13321.82 | **-14412.91** |

**Supplementary Figure 1**

Legend: Linear regression analysis of sequence divergence with time for each of the four major clusters. Genetic divergence of each sequence from the root of the inferred maximum likelihood tree was plotted against time of sampling (A). The best-fitting root was determined according to the minimized sum of the squared residuals [7] (B). Correlation coefficients ( r ) are reported.

**Supplementary Figure 2**

Legend: Relationship between TN93 distance and the difference in sample years between the pairs of directly linked NCC and LANL sequences. The TN93 distances are binned into 15 categories (0 to 0.1% is indicated by 0.1%). Thick horizontal lines: median difference in sample years for each sequence pair for a given TN93 distance bin. Upper and lower rectangle boundaries: interquartile ranges. Whiskers: the lowest and highest points within 1.5 IQR below and above the lower and upper quartiles, respectively. Circles: data points outside of this range.

**
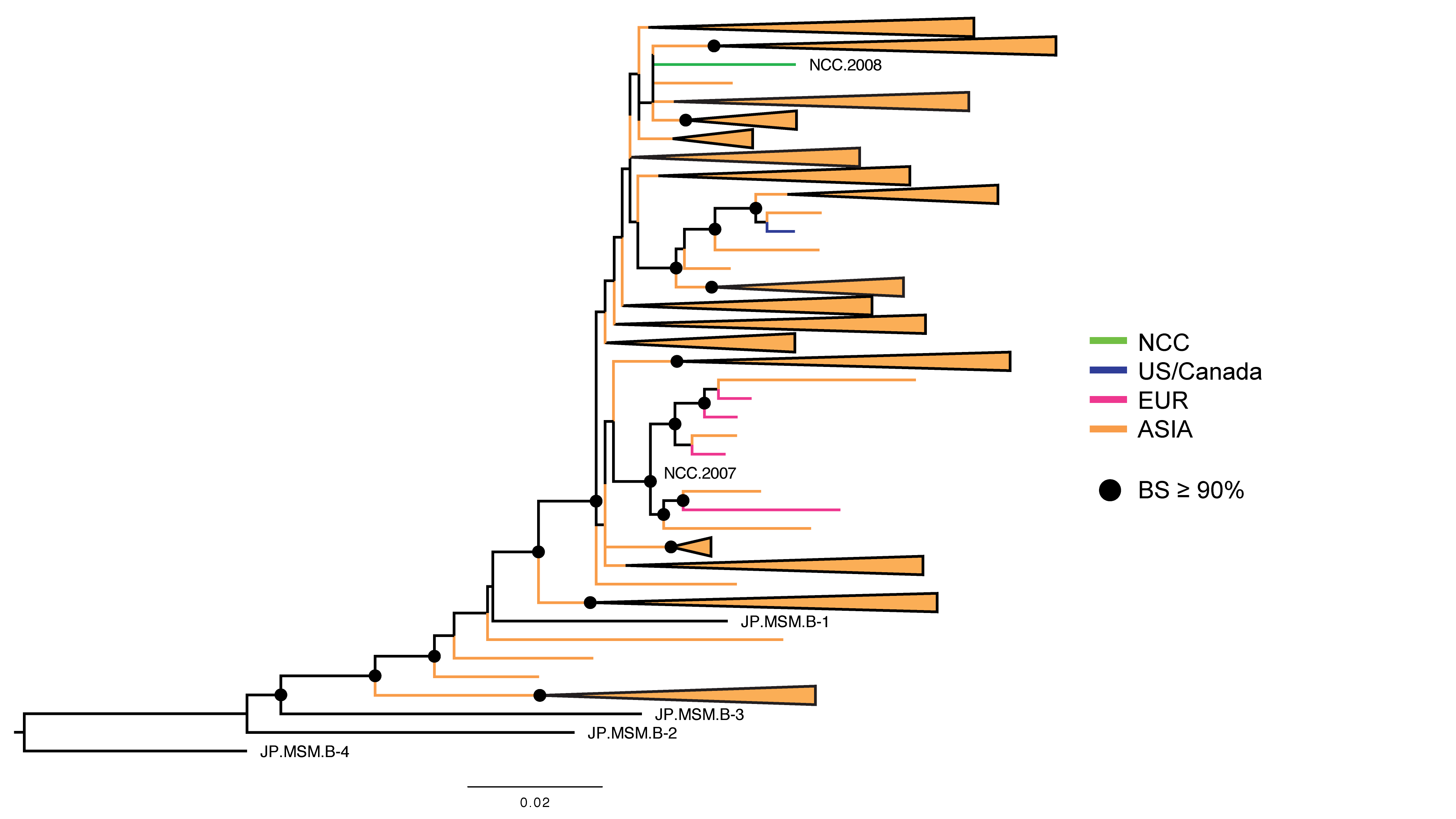
Supplementary Figure 3**

Legend: Maximum likelihood tree for the predominantly Japanese subtype B cluster and MSM reference sequences. Branch lengths are scaled in substitutions/site and are colored according to region (legend at right). The location of NCC sequences is indicated by taxon labeling. Closed circles located at interior nodes indicate bootstrap (BS) support ≥ 90% [8].

**Supplementary Figure 4**


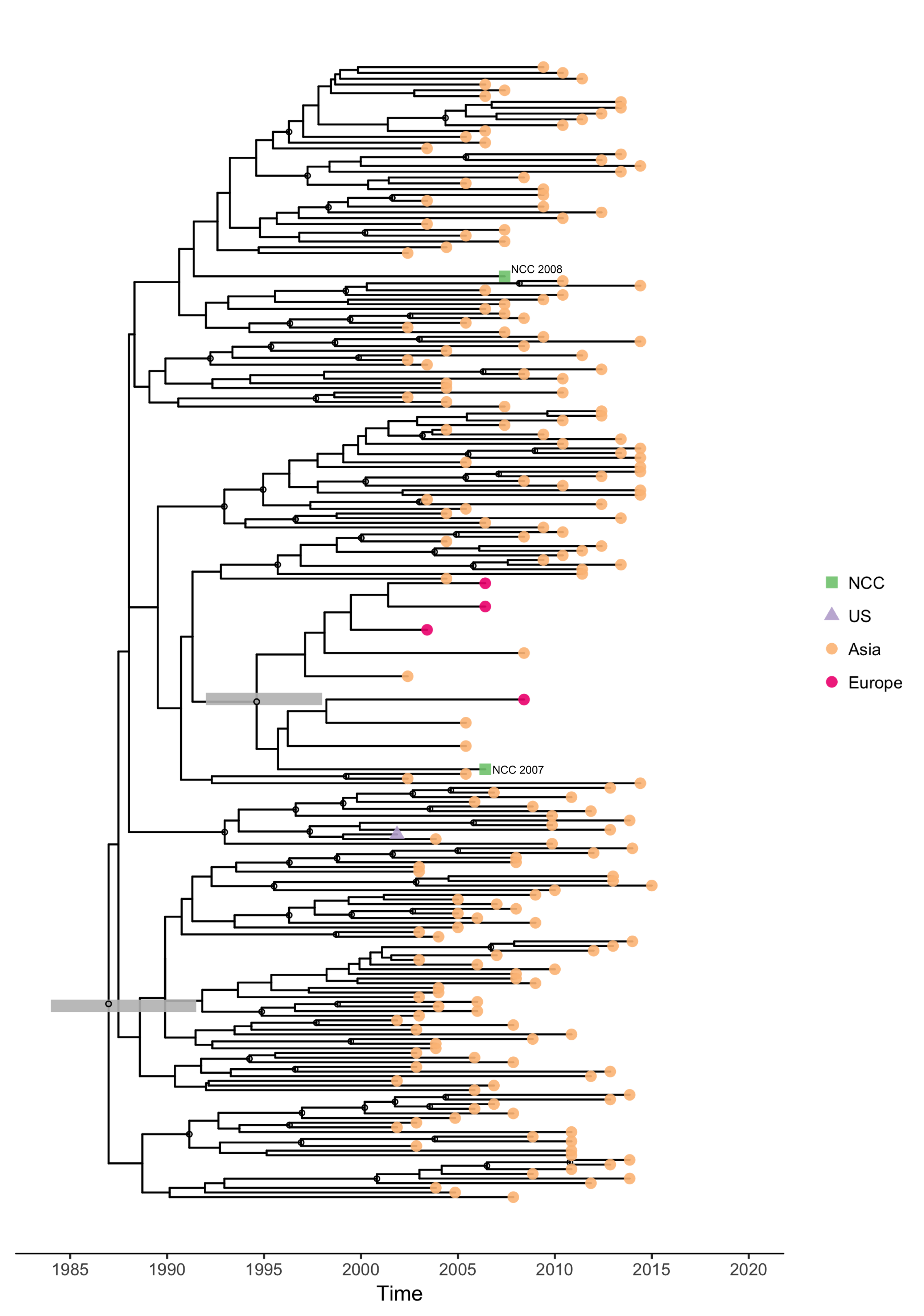


Legend: Bayesian maximum clade credibility tree for the predominantly Japanese subtype B cluster. Branch lengths are scaled in time, and tips are colored according to region of sampling (legend at right). Clades containing NCC sequences are expanded for clarity. Additionally, the location of NCC sequences is indicated by taxon labeling. Open circles located at interior nodes indicate posterior probability ≥ 90%. Bars indicate high posterior density interval estimates of the age of the most recent common ancestor of all lineages and of the diverse, global clade identified by Takebe et al. [9].

**Supplementary Figure 5**


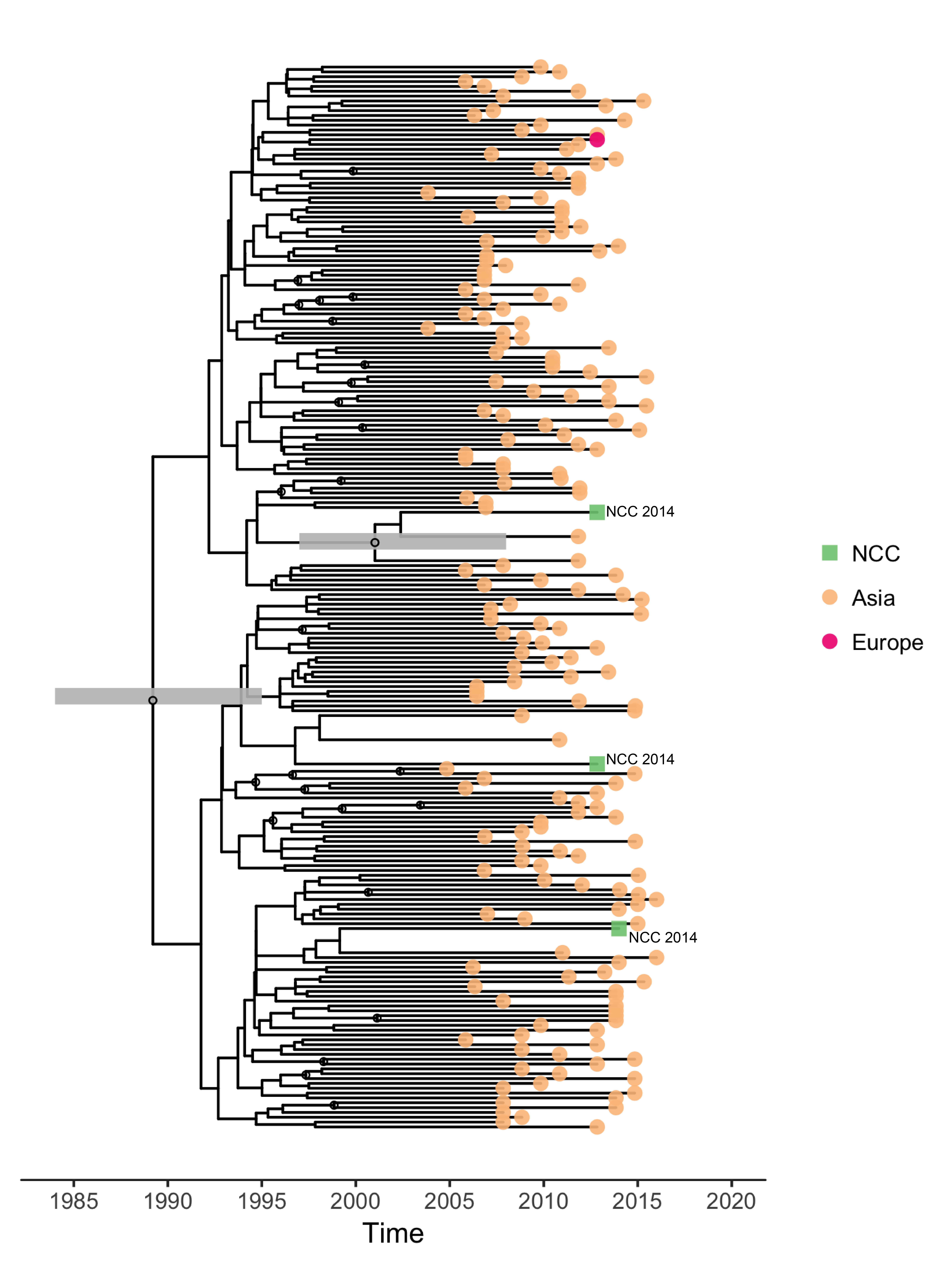


Legend: Bayesian maximum clade credibility trees for the CRF07_BC cluster. Branch lengths are scaled in time (x-axis), and tips are colored according to region of sampling (legend at right). Clades containing NCC sequences are expanded for clarity. Additionally, the location of NCC sequences is indicated by taxon labeling. Open circles located at interior nodes indicate posterior probability (PP) ≥ 90%. Bars indicate high posterior density interval estimates of the age of the most recent common ancestor of all lineages and of highly supported (PP ≥90%) clades containing NCC sequences.

**Supplementary Figure 6**


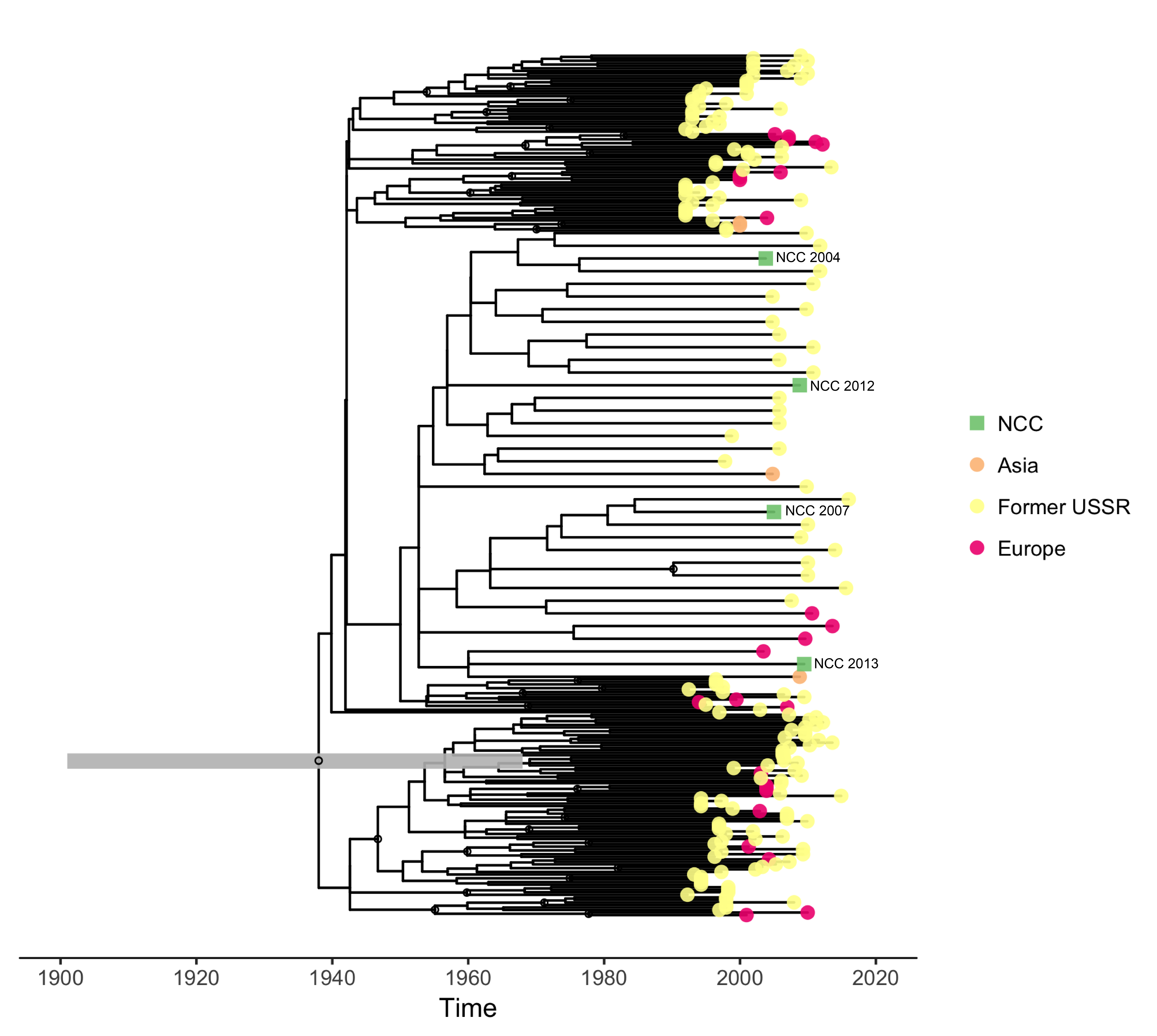


Legend: Bayesian maximum clade credibility trees for the A6 cluster. Branch lengths are scaled in time (x-axis), and tips are colored according to region of sampling (legend at right). Clades containing NCC sequences are expanded for clarity. Additionally, the location of NCC sequences is indicated by taxon labeling. Open circles located at interior nodes indicate posterior probability ≥ 90%. Bars indicate high posterior density interval estimates of the age of the most recent common ancestor of all lineages.

**Supplementary Figure 7**

Examples of small clusters that were significantly changed between NCC-only and NCC-LANL networks. Thick black outlines: NCC sequences. The country of origin (A,B) or the year of isolation (C,D) is indicated in color.

**Supplementary Figure 8**

**References for Supplementary Material**

1. Drummond AJ, Ho SY, Phillips MJ, Rambaut A. Relaxed phylogenetics and dating with confidence. PLoS Biol **2006**; 4:e88.

2. Drummond AJ, Rambaut A. BEAST: Bayesian evolutionary analysis by sampling trees. BMC Evol Biol **2007**; 7:214.

3. Drummond AJ, Suchard MA, Xie D, Rambaut A. Bayesian phylogenetics with BEAUti and the BEAST 1.7. Mol Biol Evol **2012**; 29:1969-73.

4. Baele G, Li WL, Drummond AJ, Suchard MA, Lemey P. Accurate model selection of relaxed molecular clocks in bayesian phylogenetics. Mol Biol Evol **2013**; 30:239-43.

5. Minin VN, Bloomquist EW, Suchard MA. Smooth skyride through a rough skyline: Bayesian coalescent-based inference of population dynamics. Mol Biol Evol **2008**; 25:1459-71.

6. Rambaut A, Drummond AJ. Tracer: MCMC trace analysis tool. 1.5 e. <http://treebioedacuk/software/tracer> **2009**.

7. Rambaut A, Lam TT, Max Carvalho L, Pybus OG. Exploring the temporal structure of heterochronous sequences using TempEst (formerly Path-O-Gen). Virus Evol **2016**; 2:vew007.

8. Minh BQ, Nguyen MA, von Haeseler A. Ultrafast approximation for phylogenetic bootstrap. Mol Biol Evol **2013**; 30:1188-95.

9. Takebe Y, Naito Y, Raghwani J, et al. Intercontinental dispersal of HIV-1 subtype B associated with transmission among men who have sex with men in Japan. J Virol **2014**; 88:9864-76.

**Sequence Data**

AF331679, AF331693, AF514246, AF544600, AY030645, AY030657, AY030702, AY030730, AY030731, AY030755, AY030795, AY030801, AY030803, AY030813, AY030853, AY030900, AY030905, AY030929, AY030930, AY030946, AY030952, AY031019, AY031046, AY031054, AY031079, AY031111, AY031131, AY031138, AY031150, AY031155, AY031178, AY031193, AY031204, AY031207, AY031242, AY031257, AY031279, AY031289, AY031303, AY031306, AY031326, AY031338, AY031350, AY031389, AY031406, AY031435, AY031466, AY031468, AY031483, AY031484, AY031498, AY031522, AY031552, AY031623, AY031668, AY031698, AY031723, AY031741, AY031748, AY031780, AY031829, AY031854, AY031873, AY031891, AY031928, AY031930, AY031944, AY031963, AY031977, AY032039, AY032092, AY032093, AY032106, AY032107, AY032194, AY032249, AY032256, AY032355, AY032388, AY032399, AY032441, AY032442, AY032444, AY032453, AY032462, AY032467, AY032482, AY032483, AY032509, AY032511, AY032519, AY032522, AY032553, AY032555, AY032560, AY032572, AY797481, AY797489, AY797490, AY797504, AY797532, AY797546, AY797547, AY797575, AY797576, AY797578, AY797594, AY797615, AY797619, AY797632, AY797667, AY797688, AY797703, AY797706, AY797746, AY797749, AY797781, AY797786, AY797802, AY797819, AY797827, AY797829, AY797842, AY797844, AY797857, AY797859, AY797867, AY797890, AY797908, AY797911, AY797933, AY797948, AY797953, AY797954, AY797961, AY797962, AY797975, AY797979, AY797995, AY797996, AY798001, AY798027, AY798038, AY798046, AY798058, AY798061, AY798065, AY798067, AY798073, AY798079, AY798080, AY798086, AY798090, AY798091, AY798095, AY798103, AY798107, AY798110, AY798111, AY798114, AY798115, AY798120, AY798123, AY798132, AY798138, AY798139, AY798140, AY798149, AY798153, AY798154, AY798162, AY798164, AY798170, AY798174, AY798176, AY798177, AY798179, AY798180, AY798184, AY798189, AY798191, AY798198, AY798199, AY798203, AY798204, AY798211, AY798216, AY798219, AY798225, AY798226, AY798227, AY798230, AY798236, AY798241, AY798243, AY798246, AY798247, AY798260, AY798274, AY798276, AY798279, AY798285, AY798291, AY798292, AY798293, AY798294, AY798307, AY798308, AY798312, AY798314, AY798321, AY801735, AY801743, AY801744, AY801757, AY801784, AY801797, AY801798, AY801828, AY801829, AY801832, AY801847, AY801870, AY801874, AY801887, AY801923, AY801944, AY801959, AY801962, AY802002, AY802006, AY802039, AY802044, AY802061, AY802079, AY802087, AY802089, AY802100, AY802103, AY802116, AY802118, AY802126, AY802150, AY802168, AY802171, AY802193, AY802207, AY802212, AY802213, AY802220, AY802221, AY802234, AY802238, AY802254, AY802257, AY802262, AY802288, AY802299, AY802307, AY802319, AY802322, AY802326, AY802328, AY802334, AY802339, AY802340, AY802346, AY802350, AY802351, AY802355, AY802363, AY802367, AY802370, AY802371, AY802374, AY802375, AY802380, AY802383, AY802392, AY802398, AY802399, AY802400, AY802409, AY802413, AY802414, AY802422, AY802424, AY802430, AY802434, AY802436, AY802437, AY802439, AY802440, AY802443, AY802448, AY802450, AY802457, AY802458, AY802462, AY802463, AY802470, AY802475, AY802478, AY802484, AY802485, AY802486, AY802489, AY802495, AY802500, AY802502, AY802505, AY802507, AY802520, AY802536, AY802538, AY802541, AY802547, AY802553, AY802554, AY802555, AY802556, AY802570, AY802571, AY802576, AY802578, AY802585, GQ206353, GQ206362, GQ206382, GQ206383, GQ206393, GQ206396, GQ206399, GQ206405, GQ206407, GQ206410, GQ206430, GQ206431, GQ206441, GQ206443, GQ206446, GQ206452, GQ206454, GQ206457, GQ206461, GQ206478, GQ206483, GQ206484, GQ206485, GQ206487, GQ206488, GQ206491, GQ206494, GQ206495, GQ206496, GQ206497, GQ206499, GQ206517, GQ206522, GQ206523, GQ206524, GQ206526, GQ206527, GQ206530, GQ206533, GQ206534, GQ206535, GQ206536, GQ206538, GQ206539, GQ206540, GQ206541, GQ206542, GQ206543, GQ206547, GQ206548, GQ206555, GQ206556, GQ206563, GQ206564, GQ206580, GQ206581, GQ206582, GQ206583, GQ206586, GQ206587, GQ206592, GQ206593, GQ206600, GQ206601, GQ206604, GQ206606, GQ206607, GQ206621, GQ206622, GQ206627, GQ206628, GQ206629, GQ206630, GQ206631, GQ206632, GQ206633, GQ206634, GQ206655, GQ206667, GQ206668, GQ206670, GQ206673, GQ206680, GQ206690, GQ206694, GQ206701, GQ206705, GQ206707, GQ206709, GQ206714, GQ206715, GQ206718, GQ206720, GQ206722, GQ206725, GQ206728, GQ206734, GQ206736, GQ206743, GQ206749, GQ206750, GQ206752, GQ206753, GQ206754, GQ206755, GQ206756, GQ206757, GQ206760, GQ206765, GQ206767, GQ206768, GQ206771, GQ206776, GQ206781, GQ206787, GQ206791, GQ206793, GQ206794, GQ206796, GQ206797, GQ206798, GQ206801, GQ206806, GQ206807, GQ206810, GQ206812, GQ206814, GQ206817, GQ206819, GQ206822, GQ206823, GQ206824, GQ206828, GQ206830, GQ206832, GQ206834, GQ206836, GQ206841, GQ206843, GQ206847, GQ206855, GQ206856, GQ206858, GQ206859, GQ206867, GQ206868, GQ206870, GQ206873, GQ206881, GQ206890, GQ206894, GQ206902, GQ206905, GQ206908, GQ206910, GQ206913, GQ206917, GQ206920, GQ206922, GQ206924, GQ206927, GQ206930, GQ206936, GQ206938, GQ206943, GQ206951, GQ206952, GQ206954, GQ206955, GQ206956, GQ206957, GQ206958, GQ206959, GQ206963, GQ206968, GQ206970, GQ206971, GQ206975, GQ206979, GQ206984, GQ206990, GQ206995, GQ206996, GQ206999, GQ207000, GQ207001, GQ207004, GQ207009, GQ207010, GQ207012, GQ207014, GQ207017, GQ207020, GQ207022, GQ207023, GQ207026, GQ207027, GQ207030, GQ207032, GQ207034, GQ207036, GQ207038, GQ207043, GQ207045, GQ207050, GQ207057, GQ207058, GQ207060, GQ207061, GQ207172, GQ207173, GQ207182, GQ207183, GQ207184, GQ207185, GQ207186, GQ207187, GQ207188, GQ207189, GQ207194, GQ207195, GQ207200, GQ207201, GQ207204, GQ207205, GQ207206, GQ207207, GQ207211, GQ207212, GQ207213, GQ207214, GQ207217, GQ207218, GQ207222, GQ207223, GQ207224, GQ207225, GQ207230, GQ207231, GQ207238, GQ207239, GQ207246, GQ207247, GQ207258, GQ207259, GQ207260, GQ207261, GQ207262, GQ207263, GQ207267, GQ207268, GQ207273, GQ207274, GQ207277, GQ207278, GQ207285, GQ207286, GQ207301, GQ207302, GQ207303, GQ207304, GQ207305, GQ207306, GQ207309, GQ207310, GQ207318, GQ207319, GQ207322, GQ207323, GQ207330, GQ207331, GQ207339, GQ207340, GQ207345, GQ207346, GQ207347, GQ207348, GQ207349, GQ207350, GQ207351, GQ207352, GQ207354, GQ207355, GQ207356, GQ207357, GQ207362, GQ207363, GQ207366, GQ207367, GQ207368, GQ207369, GQ207372, GQ207373, GQ207374, GQ207375, GQ207376, GQ207377, GQ207378, GQ207379, GQ207380, GQ207381, GQ207384, GQ207385, GQ207386, GQ207387, GQ207389, GQ207390, GQ207391, GQ207392, GQ207393, GQ207394, GQ207395, GQ207398, GQ207399, GQ207411, GQ207412, GQ207413, GQ207414, GQ207415, GQ207416, GQ207421, GQ207422, GQ207423, GQ207424, GQ207425, GQ207426, GQ207431, GQ207434, GQ207435, GQ207436, GQ207437, GQ207438, GQ207439, GQ207440, GQ207441, GQ207444, GQ207445, GQ207446, GQ207447, GQ207448, GQ207449, GQ207450, GQ207451, GQ207454, GQ207455, GQ207456, GQ207457, GQ207458, GQ207459, GQ207462, GQ207463, GQ207465, GQ207466, GQ207467, GQ207468, GQ207471, GQ207472, GQ207473, GQ207474, GQ207477, GQ207478, GQ207485, GQ207486, GQ207487, GQ207488, GQ207491, GQ207492, GQ207493, GQ207494, GQ207495, GQ207496, GQ207497, GQ207498, GQ207499, GQ207500, GQ207505, GQ207506, GQ207507, GQ207508, GQ207509, GQ207510, GQ207519, GQ207520, GQ207561, GQ207562, GQ207563, GQ207564, GQ207569, GQ207570, GQ207576, GQ207577, GQ207578, GQ207579, GQ207580, GQ207581, GQ207582, GQ207583, GQ207586, GQ207587, GQ207588, GQ207589, GQ207590, GQ207591, GQ207592, GQ207593, GQ207594, GQ207595, GQ207600, GQ207601, GQ207602, GQ207603, GQ207604, GQ207605, GQ207606, GQ207607, GQ207608, GQ207609, GQ207612, GQ207613, GQ207618, GQ207619, GQ207620, GQ207621, GQ207622, GQ207623, GQ207626, GQ207627, GQ207629, GQ207630, GQ207631, GQ207634, GQ207635, GQ207636, GQ207637, GQ207642, GQ207643, GQ207648, GQ207649, GQ207652, GQ207653, GQ207654, GQ207655, GQ207656, GQ207657, GQ207658, GQ207659, GQ207660, GQ207661, GQ207662, GQ207663, GQ207664, GQ207665, GQ207666, GQ207670, GQ207671, GQ207672, GQ207673, GQ207675, GQ207676, GQ207677, GQ207678, GQ207679, GQ207682, GQ207683, GQ207684, GQ207685, GQ207686, GQ207687, GQ207690, GQ207691, GQ207692, GQ207693, GQ207694, GQ207695, GQ207696, GQ207697, GQ207698, GQ207699, GQ207758, GQ207759, GQ207760, GQ207761, GQ207762, GQ207763, GQ207764, GQ207765, GQ207766, GQ207767, GQ207768, GQ207769, GQ207770, GQ207771, GQ207772, GQ207773, GQ207774, GQ207775, GQ207776, GQ207777, GQ207778, GQ207779, GQ207780, GQ207781, GQ207782, GQ207783, GQ207788, GQ207789, GQ207790, GQ207791, GQ207792, GQ207793, GQ207796, GQ207797, GQ207798, GQ207799, GQ207800, GQ207801, GQ207802, GQ207803, GQ207804, GQ207805, GQ207811, GQ207812, GQ207813, GQ207814, GQ207815, GQ207816, GQ207819, GQ207820, GQ207823, GQ207824, GQ207827, GQ207828, GQ207829, GQ207830, GQ207831, GQ207832, GQ207833, GQ207834, GQ207835, GQ207836, GQ207837, GQ207838, GQ207839, GQ207840, GQ207841, GQ207842, GQ207843, GQ207844, GQ207845, GQ207846, GQ207851, GQ207852, GQ207855, GQ207856, GQ207859, GQ207860, GQ207861, GQ207862, GQ207863, GQ207864, GQ207865, GQ207866, GQ207867, GQ207868, GQ207869, GQ207870, GQ207871, GQ207872, GQ207875, GQ207876, GQ207877, GQ207878, GQ207988, GQ207989, GQ207990, GQ207991, GQ207996, GQ207997, GQ207998, GQ207999, GQ208000, GQ208001, GQ208002, GQ208003, GQ208004, GQ208005, GQ208006, GQ208008, GQ208009, GQ208010, GQ208011, GQ208012, GQ208013, GQ208014, GQ208015, GQ208016, GQ208017, GQ208020, GQ208022, GQ208023, GQ208024, GQ208025, GQ208026, GQ208027, GQ208028, GQ208030, GQ208031, GQ208032, GQ208033, GQ208034, GQ208035, GQ208038, GQ208039, GQ208042, GQ208043, GQ208048, GQ208049, GQ208053, GQ208054, GQ208055, GQ208056, GQ208057, GQ208058, GQ208059, GQ208060, GQ208063, GQ208064, GQ208065, GQ208066, GQ208067, GQ208068, GQ208069, GQ208070, GQ208071, GQ208072, GQ208073, GQ208074, GQ208075, GQ208076, GQ208077, GQ208078, GQ208083, GQ208084, GQ208085, GQ208086, GQ208089, GQ208090, GQ208091, GQ208092, GQ208093, GQ208094, GQ208095, GQ208096, GQ208097, GQ208098, GQ208101, GQ208102, GQ208103, GQ208104, GQ208105, GQ208106, GQ208107, GQ208108, GQ208111, GQ208112, GQ208113, GQ208114, GQ208115, GQ208116, GQ208119, GQ208120, GQ208121, GQ208122, GQ208123, GQ208124, GQ208128, GQ208129, GQ208169, GQ208170, GQ208173, GQ208174, GQ208355, GQ208356, GQ208357, GQ208358, GQ208359, GQ208360, GQ208361, GQ208362, GQ208363, GQ208364, GQ208365, GQ208366, GQ208372, GQ208373, GQ208378, GQ208379, GQ208382, GQ208383, GQ208384, GQ208385, GQ208386, GQ208387, GQ208388, GQ208389, GQ208390, GQ208391, GQ208392, GQ208393, GQ208394, GQ208395, GQ208398, GQ208399, GQ208400, GQ208401, GQ208402, GQ208403, GQ208404, GQ208405, GQ208406, GQ208407, GQ208408, GQ208409, GQ208417, GQ208418, GQ208419, GQ208420, GQ208425, GQ208426, GQ208427, GQ208428, GQ208429, GQ208430, GQ208432, GQ208433, GQ208434, GQ208435, GQ208436, GQ208437, GQ208438, GQ208439, GQ208444, GQ208445, GQ208448, GQ208449, GQ208451, GQ208452, GQ208453, GQ208454, GQ208457, GQ208458, GQ208459, GQ208460, GQ208463, GQ208464, GQ208467, GQ208468, GQ208469, GQ208470, GQ208471, GQ208472, GQ208473, GQ208474, GQ208475, GQ208476, GQ208477, GQ208478, GQ208479, GQ208480, GQ208483, GQ208484, GQ208487, GQ208488, GQ208491, GQ208492, GQ208493, GQ208494, GQ208500, GQ208501, GQ208505, GQ208506, GQ208507, GQ208508, GQ208509, GQ208510, GQ208513, GQ208514, GQ208515, GQ208516, GQ208524, GQ208525, GQ208526, GQ208527, GQ208528, GQ208529, GQ208530, GQ208531, GQ208532, GQ208533, GQ208534, GQ208535, GQ208536, GQ208537, GQ208538, GQ208539, GQ208540, GQ208541, GQ208545, GQ208546, GQ208547, GQ208548, GQ208549, GQ208550, GQ208551, GQ208552, GQ208555, GQ208556, GQ208557, GQ208558, GQ208559, GQ208560, GQ208561, GQ208562, GQ208563, GQ208564, GQ208567, GQ208568, GQ208571, GQ208572, GQ208573, GQ208574, GQ208575, GQ208576, GQ208577, GQ208578, GQ208579, GQ208580, GQ208581, GQ208582, GQ208583, GQ208584, GQ208585, GQ208586, GQ208587, GQ208588, GQ208589, GQ208590, GQ208591, GQ208592, GQ208593, GQ208594, GQ208595, GQ210393, GQ210394, GQ210395, GQ210396, GQ210399, GQ210400, GQ210401, GQ210402, GQ210403, GQ210404, GQ210405, GQ210406, GQ210407, GQ210408, GQ210411, GQ210412, GQ210413, GQ210414, GQ210415, GQ210416, GQ210417, GQ210418, GQ210419, GQ210420, GQ210421, GQ210422, GQ210423, GQ210424, GQ210425, GQ210426, GQ210427, GQ210431, GQ210432, GQ210434, GQ210435, GQ210436, GQ210437, GQ210440, GQ210441, GQ210444, GQ210445, GQ210446, GQ210447, GQ210448, GQ210449, GQ210450, GQ210451, GQ210456, GQ210457, GQ210458, GQ210459, GQ210460, GQ210461, GQ210465, GQ210466, GQ210467, GQ210468, GQ210471, GQ210472, GQ210473, GQ210474, GQ210475, GQ210478, GQ210479, GQ210480, GQ210481, GQ210482, GQ210483, GQ210484, GQ210485, GQ210486, GQ210487, GQ210488, GQ210489, GQ210491, GQ210492, GQ210495, GQ210496, GQ210499, GQ210500, GQ210505, GQ210506, GQ210507, GQ210508, GQ210509, GQ210510, GQ210511, GQ210512, GQ210513, GQ210514, GQ210515, GQ210516, GQ210517, GQ210518, GQ210523, GQ210524, GQ210525, GQ210526, GQ210527, GQ210528, GQ210529, GQ210530, GQ210531, GQ210532, GQ210533, GQ210534, GQ210535, GQ210536, GQ210537, GQ210538, GQ210541, GQ210542, GQ210543, GQ210544, GQ210545, GQ210546, GQ210547, GQ210548, GQ210551, GQ210552, GQ210553, GQ210554, GQ210557, GQ210558, GQ210561, GQ210562, GQ210781, GQ210782, GQ210783, GQ210784, GQ210787, GQ210788, GQ210793, GQ210794, GQ210795, GQ210796, GQ210797, GQ210798, GQ210801, GQ210802, GQ210803, GQ210804, GQ210805, GQ210806, GQ210807, GQ210808, GQ210809, GQ210810, GQ210811, GQ210812, GQ210813, GQ210814, GQ210815, GQ210816, GQ210817, GQ210818, GQ210821, GQ210822, GQ210829, GQ210830, GQ210833, GQ210834, GQ210835, GQ210836, GQ210841, GQ210842, GQ210843, GQ210844, GQ210845, GQ210846, GQ210847, GQ210850, GQ210851, GQ210852, GQ210853, GQ210854, GQ210855, GQ210856, GQ210858, GQ210859, GQ210860, GQ210861, GQ210862, GQ210863, GQ210866, GQ210867, GQ210868, GQ210869, GQ210874, GQ210875, GQ210876, GQ210877, GQ210878, GQ210879, GQ210880, GQ210881, GQ210882, GQ210883, GQ210886, GQ210887, GQ210888, GQ210889, GQ210892, GQ210893, GQ210898, GQ210899, GQ210900, GQ210901, GQ210904, GQ210905, GQ210908, GQ210909, GQ210910, GQ210911, GQ210914, GQ210915, GQ210916, GQ210917, GQ210918, GQ210919, GQ210922, GQ210923, GQ210926, GQ210927, GQ210930, GQ210931, GQ210932, GQ210933, GQ210934, GQ210935, GQ210936, GQ210937, GQ210938, GQ210947, GQ210948, GQ210949, GQ210950, GQ210951, GQ210952, GQ210955, GQ210956, GQ210959, GQ210960, GQ210961, GQ210962, GQ210963, GQ210966, GQ210967, GQ210968, GQ210969, GQ210971, GQ210972, GQ210973, GQ210974, GQ210975, GQ210976, GQ210979, GQ210980, GQ210983, GQ210984, GQ210986, GQ210987, GQ210990, GQ210991, GQ210992, GQ210993, GQ210994, GQ210995, GQ210996, GQ210997, GQ210998, GQ210999, GQ211002, GQ211003, GQ211005, GQ211006, GQ211011, GQ211012, GQ211013, GQ211014, GQ211017, GQ211018, GQ211019, GQ211020, GQ211021, GQ211022, GQ211023, GQ211024, GQ211025, GQ211026, GQ211030, GQ211031, GQ211032, GQ211033, GQ211034, GQ211035, GQ211040, GQ211041, GQ211043, GQ211044, GQ211045, GQ211046, GQ211047, GQ211048, GQ211049, GQ211052, GQ211053, GQ211054, GQ211055, GQ211056, GQ211057, GQ211058, GQ211059, GQ211060, GQ211061, GQ211064, GQ211065, GQ211066, GQ211067, GQ211068, GQ211069, GQ211070, GQ211071, GQ211072, GQ211073, GQ211074, GQ211075, GQ211076, GQ211077, GQ211078, GQ211079, GQ211080, GQ211081, GQ211082, GQ211083, GQ211084, GQ211085, GQ211086, GQ211087, GQ211088, GQ211089, GQ211094, GQ211095, GQ211096, GQ211097, GQ211104, GQ211105, GQ211106, GQ211107, GQ211225, GQ211226, GQ211227, GQ211228, GQ211229, GQ211230, GQ211233, GQ211234, GQ211235, GQ211236, GQ211237, GQ211238, GQ211239, GQ211240, GQ211242, GQ211243, GQ211244, GQ211245, GQ211246, GQ211247, GQ211250, GQ211251, GQ211252, GQ211253, GQ211254, GQ211255, GQ211256, GQ211257, GQ211258, GQ211259, GQ211262, GQ211263, GQ211264, GQ211265, GQ211266, GQ211267, GQ211268, GQ211269, GQ211272, GQ211273, GQ211274, GQ211275, GQ211278, GQ211279, GQ211282, GQ211283, GQ211284, GQ211285, GQ211288, GQ211289, GQ211294, GQ211295, GQ211296, GQ211297, GQ211298, GQ211299, GQ211306, GQ211307, GQ211308, GQ211309, GQ211310, GQ211311, GQ211312, GQ211313, GQ211315, GQ211316, GQ211317, GQ211318, GQ211319, GQ211320, GQ211321, GQ211322, GQ211323, GQ211324, GQ211328, GQ211329, GQ211330, GQ211331, GQ211332, GQ211333, GQ211334, GQ211337, GQ211338, GQ211339, GQ211340, GQ211341, GQ211342, GQ211347, GQ211348, GQ211349, GQ211350, GQ211351, GQ211352, GQ211356, GQ211357, GQ211361, GQ211362, GQ211363, GQ211364, GQ211365, GQ211366, GQ211371, GQ211372, GQ211373, GQ211374, GQ211386, GQ211387, GQ211388, GQ211389, GQ211390, GQ211391, GQ211396, GQ211397, GQ211402, GQ211403, GQ211406, GQ211407, GQ211539, GQ211540, GQ211541, GQ211542, GQ211543, GQ211544, GQ211547, GQ211548, GQ211549, GQ211550, GQ211553, GQ211554, GQ211555, GQ211556, GQ211557, GQ211558, GQ211559, GQ211560, GQ211561, GQ211562, GQ211563, GQ211564, GQ211565, GQ211566, GQ211567, GQ211568, GQ211569, GQ211570, GQ211573, GQ211574, GQ211575, GQ211576, GQ211577, GQ211578, GQ211579, GQ211580, GQ211581, GQ211582, GQ211583, GQ211584, GQ211585, GQ211586, GQ211587, GQ211588, GQ211589, GQ211590, GQ211593, GQ211594, GQ211595, GQ211596, GQ211599, GQ211600, GQ211601, GQ211602, GQ211603, GQ211604, GQ211607, GQ211608, GQ211609, GQ211610, GQ211611, GQ211612, GQ211613, GQ211614, GQ211615, GQ211616, GQ211621, GQ211622, GQ211623, GQ211624, GQ211625, GQ211626, GQ211627, GQ211630, GQ211631, GQ211632, GQ211633, GQ211634, GQ211635, GQ211636, GQ211637, GQ211638, GQ211639, GQ211640, GQ211641, GQ211647, GQ211649, GQ211650, GQ211657, GQ211658, GQ211662, GQ211663, GQ211664, GQ211665, GQ211666, GQ211667, GQ211668, GQ211669, GQ211670, GQ211671, GQ211674, GQ211675, GQ211676, GQ211677, GQ211678, GQ211679, GQ211680, GQ211681, GQ211684, GQ211685, GQ211686, GQ211687, GQ211688, GQ211689, GQ211690, GQ211691, GQ211692, GQ211693, GQ211694, GQ211695, GQ211696, GQ211697, GQ211699, GQ211700, GQ211701, GQ211702, GQ211703, GQ211704, GQ211705, GQ211706, GQ211707, GQ211708, GQ211709, GQ211710, GQ211711, GQ211712, GQ211713, GQ211715, GQ211716, GQ211718, GQ211719, GQ211720, GQ211721, GQ211724, GQ211725, GQ211726, GQ211727, GQ211730, GQ211731, GQ211732, GQ211733, GQ211734, GQ211735, GQ211738, GQ211739, GQ211740, GQ211741, GQ211742, GQ211743, GQ211744, GQ211745, GQ211746, GQ211747, GQ211748, GQ211751, GQ211752, GQ211753, GQ211754, GQ211760, GQ211761, GQ211936, GQ211937, GQ211938, GQ211939, GQ211940, GQ211941, GQ211943, GQ211944, GQ211945, GQ211946, GQ211947, GQ211948, GQ211949, GQ211950, GQ211953, GQ211954, GQ211955, GQ211956, GQ211957, GQ211958, GQ211959, GQ211960, GQ211966, GQ211967, GQ211968, GQ211969, GQ211970, GQ211971, GQ211972, GQ211973, GQ211974, GQ211975, GQ211978, GQ211979, GQ211981, GQ211982, GQ211983, GQ211984, GQ211985, GQ211986, GQ211987, GQ211988, GQ211989, GQ211990, GQ211991, GQ211992, GQ211995, GQ211996, GQ211999, GQ212000, GQ212001, GQ212002, GQ212003, GQ212004, GQ212007, GQ212008, GQ212011, GQ212012, GQ212013, GQ212014, GQ212015, GQ212016, GQ212017, GQ212018, GQ212019, GQ212020, GQ212021, GQ212022, GQ212023, GQ212024, GQ212025, GQ212026, GQ212027, GQ212028, GQ212029, GQ212030, GQ212031, GQ212032, GQ212033, GQ212034, GQ212035, GQ212036, GQ212037, GQ212038, GQ212039, GQ212040, GQ212041, GQ212042, GQ212043, GQ212044, GQ212045, GQ212046, GQ212047, GQ212048, GQ212049, GQ212050, GQ212051, GQ212054, GQ212055, GQ212056, GQ212057, GQ212058, GQ212059, GQ212062, GQ212063, GQ212064, GQ212065, GQ212066, GQ212067, GQ212068, GQ212069, GQ212070, GQ212071, GQ212072, GQ212073, GQ212076, GQ212077, GQ212080, GQ212081, GQ212084, GQ212085, GQ212086, GQ212087, GQ212090, GQ212091, GQ212092, GQ212093, GQ212094, GQ212095, GQ212096, GQ212097, GQ212098, GQ212099, GQ212100, GQ212101, GQ212103, GQ212104, GQ212105, GQ212106, GQ212107, GQ212108, GQ212109, GQ212110, GQ212113, GQ212114, GQ212115, GQ212116, GQ212119, GQ212120, GQ212122, GQ212123, GQ212128, GQ212129, GQ212130, GQ212131, GQ212132, GQ212133, GQ212134, GQ212135, GQ212138, GQ212139, GQ212140, GQ212141, GQ212142, GQ212143, GQ212144, GQ212145, GQ212146, GQ212147, GQ212148, GQ212149, GQ212154, GQ212155, GQ212156, GQ212157, GQ212158, GQ212159, GQ212160, GQ212165, GQ212166, GQ212167, GQ212168, GQ212169, GQ212172, GQ212173, GQ212174, GQ212175, GQ212177, GQ212178, GQ212179, GQ212180, GQ212181, GQ212182, GQ212183, GQ212184, GQ212185, GQ212186, GQ212187, GQ212188, GQ212189, GQ212190, GQ212191, GQ212192, GQ212195, GQ212196, GQ212197, GQ212198, GQ212199, GQ212200, GQ212203, GQ212204, GQ212205, GQ212206, GQ212207, GQ212208, GQ212209, GQ212210, GQ212211, GQ212212, GQ212213, GQ212214, GQ212215, GQ212216, GQ212217, GQ212218, GQ212219, GQ212220, GQ212221, GQ212222, GQ212223, GQ212224, GQ212225, GQ212226, GQ212227, GQ212228, GQ212229, GQ212230, GQ212231, GQ212232, GQ212233, GQ212234, GQ212235, GQ212236, GQ212237, GQ212238, GQ212239, GQ212240, GQ212241, GQ212242, GQ212243, GQ212244, GQ212247, GQ212248, GQ212249, GQ212250, GQ212251, GQ212252, GQ212253, GQ212254, GQ212255, GQ212256, GQ212257, GQ212258, GQ212259, GQ212260, GQ212261, GQ212262, GQ212265, GQ212270, GQ212271, GQ212274, GQ212275, GQ212276, GQ212277, GQ212280, GQ212281, GQ212282, GQ212283, GQ212284, GQ212285, GQ212286, GQ212287, GQ212450, GQ212451, GQ212452, GQ212453, GQ212454, GQ212455, GQ212456, GQ212457, GQ212458, GQ212459, GQ212461, GQ212462, GQ212465, GQ212466, GQ212467, GQ212468, GQ212469, GQ212470, GQ212471, GQ212472, GQ212475, GQ212476, GQ212479, GQ212480, GQ212481, GQ212482, GQ212485, GQ212486, GQ212487, GQ212488, GQ212489, GQ212490, GQ212491, GQ212492, GQ212493, GQ212494, GQ212495, GQ212496, GQ212497, GQ212498, GQ212499, GQ212500, GQ212501, GQ212502, GQ212503, GQ212504, GQ212505, GQ212506, GQ212507, GQ212508, GQ212511, GQ212512, GQ212518, GQ212519, GQ212520, GQ212521, GQ212522, GQ212523, GQ212524, GQ212525, GQ212528, GQ212529, GQ212530, GQ212531, GQ212532, GQ212533, GQ212534, GQ212535, GQ212536, GQ212537, GQ212538, GQ212539, GQ212540, GQ212541, GQ212542, GQ212543, GQ212544, GQ212545, GQ212546, GQ212547, GQ212548, GQ212549, GQ212550, GQ212551, GQ212552, GQ212553, GQ212554, GQ212555, GQ212556, GQ212557, GQ212558, GQ212561, GQ212562, GQ212563, GQ212564, GQ212565, GQ212566, GQ212567, GQ212568, GQ212571, GQ212572, GQ212573, GQ212574, GQ212575, GQ212576, GQ212577, GQ212578, GQ212580, GQ212581, GQ212584, GQ212585, GQ212586, GQ212587, GQ212588, GQ212589, GQ212590, GQ212591, GQ212592, GQ212593, GQ212594, GQ212595, GQ212596, GQ212597, GQ212600, GQ212601, GQ212604, GQ212605, GQ212606, GQ212607, GQ212608, GQ212609, GQ212610, GQ212613, GQ212614, GQ212615, GQ212616, GQ212620, GQ212621, GQ212626, GQ212627, GQ212628, GQ212629, GQ212630, GQ212631, GQ212632, GQ212633, GQ212636, GQ212637, GQ212640, GQ212641, GQ212642, GQ212643, GQ212644, GQ212645, GQ212646, GQ212647, GQ212650, GQ212651, GQ212654, GQ212655, GQ212658, GQ212659, GQ212660, GQ212661, GQ212662, GQ212663, GQ212664, GQ212665, GQ212666, GQ212667, GQ212668, GQ212669, GQ212670, GQ212671, GQ212672, GQ212673, GQ212674, GQ212675, GQ212676, GQ212677, GQ212678, GQ212679, GQ212680, GQ212681, GQ212684, GQ212685, GQ212686, GQ212687, GQ212688, GQ212689, GQ212690, GQ212691, GQ212692, GQ212693, GQ212696, GQ212697, GQ212698, GQ212699, GQ212700, GQ212701, GQ212702, GQ212703, GQ212704, GQ212705, GQ212706, GQ212707, GQ212708, GQ212709, GQ212710, GQ212711, GQ212712, GQ212713, GQ212716, GQ212717, GQ212718, GQ212719, GQ212720, GQ212721, GQ212851, GQ212852, GQ212853, GQ212854, GQ212855, GQ212856, GQ212857, GQ212858, GQ212859, GQ212860, GQ212861, GQ212862, GQ212863, GQ212864, GQ212865, GQ212866, GQ212867, GQ212868, GQ212869, GQ212870, GQ212871, GQ212872, GQ212873, GQ212874, GQ212877, GQ212878, GQ212879, GQ212880, GQ212881, GQ212882, GQ212883, GQ212884, GQ212885, GQ212886, GQ212887, GQ212888, GQ212889, GQ212890, GQ212891, GQ212892, GQ212893, GQ212894, GQ212897, GQ212898, GQ212899, GQ212900, GQ212901, GQ212902, GQ212903, GQ212904, GQ212905, GQ212906, GQ212907, GQ212908, GQ212909, GQ212910, GQ212911, GQ212912, GQ212913, GQ212914, GQ212915, GQ212916, GQ212917, GQ212918, GQ212919, GQ212920, GQ212923, GQ212924, GQ212929, GQ212930, GQ212932, GQ212933, GQ212934, GQ212935, GQ212938, GQ212939, GQ212940, GQ212941, GQ212942, GQ212943, GQ212944, GQ212949, GQ212950, GQ212953, GQ212954, GQ212955, GQ212956, GQ212957, GQ212958, GQ212965, GQ212966, GQ212968, GQ212969, GQ212970, GQ212971, GQ212972, GQ212973, GQ212974, GQ212975, GQ212976, GQ212977, GQ212978, GQ212979, GQ212983, GQ212984, GQ212987, GQ212988, GQ212989, GQ212990, GQ212993, GQ212994, GQ212995, GQ212996, GQ212997, GQ212998, GQ213005, GQ213006, GQ213009, GQ213010, GQ213013, GQ213014, GQ213203, GQ213204, GQ213207, GQ213208, GQ213215, GQ213216, GQ213217, GQ213218, GQ213219, GQ213220, GQ213221, GQ213222, GQ213223, GQ213224, GQ213225, GQ213226, GQ213229, GQ213230, GQ213233, GQ213234, GQ213235, GQ213236, GQ213237, GQ213238, GQ213243, GQ213244, GQ213245, GQ213246, GQ213247, GQ213248, GQ213249, GQ213250, GQ213251, GQ213252, GQ213253, GQ213254, GQ213255, GQ213258, GQ213259, GQ213260, GQ213261, GQ213262, GQ213263, GQ213266, GQ213267, GQ213268, GQ213271, GQ213272, GQ213275, GQ213276, GQ213277, GQ213278, GQ213279, GQ213280, GQ213369, GQ213370, GQ213407, GQ213408, GQ213409, GQ213415, GQ213416, GQ213575, GQ213576, GQ213579, GQ213580, GQ213581, GQ213582, GQ213591, GQ213592, GQ213593, GQ213594, GQ213595, GQ213596, GQ213691, GQ213692, JN940748, JN940749, JN940750, JN940751, JN940752, JN940753, JN940754, JN940755, JN940756, JN940757, JN940758, JN940759, JN940760, JN940761, JN940762, JN940763, JN940764, JN940765, JN940766, JN940767, JN940768, JN940769, JN940770, JN940771, JN940772, JN940773, JN940774, JN940775, JN940776, JN940777, JN940778, JN940779, JN940781, JN940782, JN940783, KT340005, KT340007, KT340009, KT340014, KT340020, KT340024, KT340032, KT340034, KT340036, KT340041, KT340047, KT340051, KY190192, KY190196, KY190212, KY586150, KY586151, KY586152, KY586153, KY586154, KY586155, KY586156, KY586157, KY586159, KY586160, KY586162, KY586163, KY586164, KY586165, KY586166, KY586167, KY586168, KY586169, KY586170, KY586171, KY586172, KY586173, KY586174, KY586175, KY586176, KY586177, KY586178, KY586179, KY586180, KY586181, KY586182, KY586183, KY586184, KY586185, KY586186, KY586187, KY586188, KY586189, KY586190, KY586191, KY586192, KY586193, KY586194, KY586195, KY586196, KY586197, KY586198, KY586199, KY586200, KY586201, KY586202, KY586203, KY586204, KY586205, KY586206, KY586207, KY586208, KY586209, KY586210, MG171047, MG171048, MG171050, MG171051, MG171053, MG171055, MG171056, MG171058, MG941022 - MG944215
